# Chemometric Study of the Ex Situ Underground Coal Gasification Wastewater Experimental Data

**DOI:** 10.1007/s11270-012-1311-5

**Published:** 2012-09-22

**Authors:** Adam Smoliński, Krzysztof Stańczyk, Krzysztof Kapusta, Natalia Howaniec

**Affiliations:** Department of Energy Saving and Air Protection, Central Mining Institute, Plac Gwarków 1, 40-166 Katowice, Poland

**Keywords:** Coal, Chemometrics, Wastewater, Groundwater, Underground gasification

## Abstract

The main goal of the study was the analysis of the parameters of wastewater generated during the ex situ underground coal gasification (UCG) experiments on lignite from Belchatow, and hard coal from Ziemowit and Bobrek coal mines, simulated in the ex situ reactor. The UCG wastewater may pose a potential threat to the groundwater since it contains high concentrations of inorganic (i.e., ammonia nitrogen, nitrites, chlorides, free and bound cyanides, sulfates and trace elements: As, B, Cr, Zn, Al, Cd, Co, Mn, Cu, Mo, Ni, Pb, Hg, Se, Ti, Fe) and organic (i.e., phenolics, benzene and their alkyl derivatives, and polycyclic aromatic hydrocarbons) contaminants. The principal component analysis and hierarchical clustering analysis enabled to effectively explore the similarities and dissimilarities between the samples generated in lignite and hard coal oxygen gasification process in terms of the amounts and concentrations of particular components. The total amount of wastewater produced in lignite gasification process was higher than the amount generated in hard coal gasification experiments. The lignite gasification wastewater was also characterized by the highest contents of acenaphthene, phenanthrene, anthracene, fluoranthene, and pyrene, whereas hard coal gasification wastewater was characterized by relatively higher concentrations of nitrites, As, Cr, Cu, benzene, toluene, xylene, benzo(*a*)anthracene, chrysene, benzo(*b*)fluoranthene, benzo(*k*)fluoranthene, and benzo(*a*)pyrene.

## Introduction

The underground coal gasification (UCG) is considered to be a prospective technique of coal utilization for the purposes of energy generation and chemical synthesis substrates production. Its advantages over a traditional coal gasification process results from the utilization of coal in situ and includes avoidance of cost and/or risk generating operational steps, like involvement of manpower underground, coal transport and pre-processing at the surface, as well as surface gasification infrastructure. It also enables to utilize coal resources otherwise abandoned for technical or economical reasons. Therefore, it is of special interest to Poland with the energy sector based predominantly on coal (Białecka 2008; Kapusta and Stańczyk 2009).

The first large-scale UCG trials date back to the 1930s of the twentieth century (former Soviet Union). Since then, a few dozen large-scale experiments and commercial applications of the UCG process have been accomplished (Gregg and Edgar 1978; Burton et al. 2006). Most of them were focused on technological aspects, with less attention given to the environmental issues related to the process. The results of the extensive studies on the potential groundwater pollution resulting from the UCG operation based on Hanna and Hoe Creek experience (Wyoming, USA) have been reported in the 1980s and 1990s (Stuermer et al. 1982; Lindblom 1992; Lindblom and Smith 1993; Covell and Thomas 1996). The environmental aspects have also been paid much attention in Chinchilla project (Australia) (Blinderman 2005). The latest news on Kingaroy project (Queensland, Australia) also proves that the environmental aspects of the UCG process should not be neglected (Burgess 2011; Sollars 2011).

In the previous works, the technological aspects of the UCG process performed under the ex situ and in situ conditions in Poland were described (Stańczyk et al. 2010; 2011; 2012). The issue of the groundwater pollution and its dependence on the kind of coal used and operating parameters applied was also examined (Kapusta and Stańczyk 2011). In the paper presented, an attempt of application of chemometric methods in the more in-depth analysis of the relationships between coal types, sampling time and various water contaminants generated in the oxygen blown UCG process simulated in ex situ reactors was made.

## Materials and Methods

The studied experimental data included measurements of physical and chemical parameters in wastewater generated in the UCG process on lignite and hard coal simulated in the ex situ reactor. The chemometric methods, such as the principal component analysis (PCA) and the hierarchical clustering analysis (HCA), were used in effective exploration of the studied experimental data.

### Experimental Procedure of Underground Coal Gasification in the Ex Situ Reactor

**Fig. 1:**
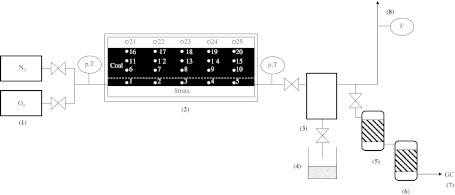
Scheme of the installation for the ex situ UCG experiments: (*1*) gasification agents and inert gas inlets, (*2*) ex situ reactor, (*3*) separator with waste water collector and (*4*) sampling point, (*5*) dehumidifier, (*6*) solid particles filter, (*7*) gas chromatograph and (*8*) gas flow meter

The experiments of lignite and hard coal underground gasification were performed in the ex situ reactor. The in situ conditions of the UCG process were simulated both in terms of coal seam and the surrounding rock mass. The UCG ex situ reactor was designed in the shape of rectangular prism of the internal workspace dimensions of: 2.6 × 1.0 × 1.1 m. Lignite or hard coal blocks were placed inside the strata layers, composed of sand collected from the site of lignite and hard coal blocks extraction and grinded clay slates. The scheme of the ex situ reactor is presented in Fig. 1. In the bottom part of the reactor, the gasification channel of the dimensions of 0.1 × 0.1 m was drilled. The numbers 1–20 and 21–25 denote the thermocouples located in a coal seam and strata, respectively.

Lignite and hard coal blocks were ignited with a specially designed gas burner fuelled with a propane–butane gas and oxygen as the oxidant. The ignition step was terminated after approximately 2 h, when the temperature in the gasification channel exceeded 400 °C. Next, the gas burner was replaced with the gasification agents feeding system and the gasification process was started. The duration of the gasification experiments presented in the study was 50 h. The process was carried out under ambient pressure with oxygen as the gasification agent (flow rate of 3–6 m^3^/h). Exothermic reactions of coal with oxygen enabled heating up the coal seam and accumulation of the sufficient amount of thermal energy for the endothermic gasification reactions. The amount of the gaseous product and its composition were continuously measured with the flow meter and the two-channel gas chromatograph Agilent 3000A, respectively. The wastewater was collected in the separator and sampled every 12 h of the experiments duration.

### Coal Blocks for UCG Tests

The block of lignite (BL) was supplied by the open cast Bełchatów (PGE KWB Bełchatów SA), and two blocks of hard coal, ZHC and BHC, were provided by Ziemowit and Bobrek coal mines (Kompania Węglowa SA), respectively. The samples were analyzed in the accredited Laboratory of Solid Fuels Quality Assessment of the Central Mining Institute in accordance with the relevant standards in force: PN-G-04511:1980 (total moisture), PN-G-04560:1998 and PN-ISO 1171:2002 (ash), PN-G-04516:1998 and PN-ISO 562:2000 (volatiles), PN-G-04513:1981 (heat of combustion, calorific value), PN-G-04584:2001 and PN-ISO 334:1997 (total sulfur), PN-G-04571:1998 (carbon, hydrogen, and nitrogen content), PN-G-04516:1998 (fixed carbon calculated as 100 − total moisture − ash − volatiles) (see Table 1).

**Table 1:** Proximate and ultimate analyses of tested lignite and hard coal samples

Parameter/coal sample	BL	ZHC	BHC
As-received			
1. Total moisture W^r^ [%]	53.0	9.0	4.0
2. Ash A^r^ [%]	4.7	5.4	10.0
3. Total sulfur S_t_^r^ [%]	1.1	0.6	1.0
4. Calorific value Q_i_^r^ [kJ/kg]	9,316	26,969	28,611
Analytical			
1. Total moisture W^a^ [%]	15.5	6.8	2.1
2. Ash A^a^ [%]	8.6	5.5	10.2
3. Volatiles V^a^ [%]	42.8	35.9	33.2
4. Heat of combustion Q_s_^a^ [kJ/kg]	20,161	28,782	30,327
5. Calorific value Q_i_^a^ [kJ/kg]	18,955	27,581	29,242
6. Total sulfur S_t_^a^ [%]	1.9	0.6	1.0
7. Carbon C_t_^a^ [%]	50.7	69.6	73.6
8. Hydrogen H_t_^a^ [%]	3.9	4.6	4.7
9. Nitrogen N^a^ [%]	1.3	0.9	1.2

### UCG Wastewater

The data set studied included measurements of 46 different physical and chemical parameters (listed in Table 2) measured in 12 wastewater samples collected after the 12th, 24th, 36th, and 48th hour of the ex situ UCG experiments duration. The samples were filtered in order to remove coal chars and other solids and analyzed, using standard analytical methods, in the accredited Laboratory of Water and Sewage Analysis of the Central Mining Institute. The electrochemical methods, such as potentiometry and conductometry were applied to measure pH and the conductivity of the wastewater, respectively. The flow injection analysis with gaseous diffusion and spectrophotometric detection and segmented flow analysis with spectrophotometric detection were applied to measure the ammonia nitrogen and cyanides concentrations, respectively. The sulfates were precipitated with barium prior to the determination of their concentration with a gravimetric method. The amount of boron and other metals were determined by inductively coupled plasma-optical emission spectroscopy. Benzene and their alkyl derivatives (benzene, toluene, ethyl benzene, and xylene—BTEX) concentrations were assessed with the gas chromatography headspace method coupled with a mass spectrometry. High-performance liquid chromatography was applied to determine the polycyclic aromatic hydrocarbons (PAHs) content.

**Table 2:** Chemical and physical parameters analyzed for the ex situ UCG wastewater

No	Parameters	Units
1	Conductivity	μS/cm
2	pH	pH
3	Ammonia nitrogen	mg/l
4	Suspended matter	mg/l
5	Nitrites	mg/l
6	Chlorides	mg/l
7	Total cyanides	mg/l
8	Free cyanides	mg/l
9	Bound cyanides	mg/l
10	Phenols	mg/l
11	Sulfates	mg/l
12	Total amount of chlorides and sulfates	mg/l
13	Ammonium	mg/l
14	Antimony	mg/l
15	Arsenic	mg/l
16	Boron	mg/l
17	Chromium	mg/l
18	Zinc	mg/l
19	Aluminum	mg/l
20	Cadmium	mg/l
21	Cobalt	mg/l
22	Manganese	mg/l
23	Copper	mg/l
24	Molybdenum	mg/l
25	Nickel	mg/l
26	Lead	mg/l
27	Mercury	mg/l
28	Selenium	mg/l
29	Titanium	mg/l
30	Iron	mg/l
31	Benzene	μg/l
32	Toluene	μg/l
33	Ethyl benzene	μg/l
34	Xylene	μg/l
35	Naphthalene	μg/l
36	Acenaphthene	μg/l
37	Fluorene	μg/l
38	Phenanthrene	μg/l
39	Anthracene	μg/l
40	Fluoranthene	μg/l
41	Pyrene	μg/l
42	Benzo(*a*) anthracene	μg/l
43	Chrysene	μg/l
44	Benzo(*b*)fluoranthene	μg/l
45	Benzo(*k*)fluoranthene	μg/l
46	Benzo(*a*)pyrene	μg/l

The studied UCG wastewater data set was organized in the matrix **X** (12 × 46). The rows and the columns of the matrix **X** (12 × 46) represent the wastewater samples collected after the 12th, 24th, 36th, and 48th hour of the experiments of BL, ZHC and BHC gasification duration, and the 46 measured parameters, respectively. Since the measured parameters differed in their ranges, the studied data set **X** (12 × 46) was standardized, according to the formula:

1 $$ {x_{jk}} = \frac{{\left( {{x_{jk}} - {{\overline{x}}_k}} \right)}}{{{s_k}}} $$

where ${\overline x_k}$ and *S*_*k*_ denote the mean of the *j*th column and its standard deviation, respectively. The standardized data matrix is denoted as **X**_c_ (12 × 46).

### Methods of Exploratory Analysis of the Ex Situ UCG Process Wastewater Data set

The simplest way of tracing the relationships between the analyzed samples is their visualization in the space of measured parameters. Since the analyzed wastewater data would require 46-dimension space (46 parameters measured for each wastewater sample), the reduction of the data dimensionality was needed. The PCA (Joliffe 1986; Wold 1987; Massart et al. 1997; Vandeginste et al. 1998) is one of the methods most commonly used for reduction of data dimensionality. This method, provided that the reduction of data dimensionality is effective, enables data visualization and partial reduction of experimental error. PCA results in a decomposition of a data matrix **X** (*m* × *n*), into matrices **S** (*m* × *A*), and **D** (*A* × *n*), where *A* denotes the number of significant factors:

2 $$ {{\bf X}}\left( {m \times n} \right) = {{\bf S}}\left( {m \times A} \right) \times {{\bf D}}\left( {A \times n} \right) + {{\bf E}}\left( {m \times n} \right) $$

The matrix **S** is called a score matrix and includes, in case of the data set considered, information on wastewater sampled during three ex situ UCG experiments performed on lignite (1 trial) and hard coal (2 trials) in the 12th, 24th, 36th, and 48th hour of each experiment duration. The matrix **D**, called a loading matrix, describes the measured parameters. Columns of matrix **S** and rows of matrix **D** are called principal components (PCs) or eigenvectors and are constructed in a way maximizing description of the data variance. The way of construction of consecutive PCs is enforced by their orthogonality and their arrangement according to decreasing data variance they describe. Since the principal components provide the information not only on the data structure but also on the experimental error, the optimal number of the principal components (A) need to be selected, enabling effective data modeling and simultaneously, partial elimination of experimental error. The matrix **E** includes the part of data, which has not been modeled by the constructed model with A PCs. The problem of ineffective data dimension reduction in PCA may be resolved with a use of the HCA (Ward 1963; Massart and Kaufman 1983; Romesburg 1984; Vogt et al. 1987; Kaufman and Rousseeuw 1990; Vandeginste et al. 1998).

The HCA enables analysis of data structure by tracing the similarities between objects in the parameters space and parameters in the objects space. The HCA methods differ in terms of parameters similarity measure used and ways of cluster linking. The results are presented in a form of dendrograms, of which OX axis shows the sequence of objects/parameters clustering and OY axis determines the similarity between them. The HCA does not enable parallel tracing of similarities between objects and measured parameters. In 2002, Smoliński et al. (2002) proposed a method of solving this problem by complementing HCA with a color map of experimental data, which enabled more in-depth interpretation of data structure.

## Results and Discussion

The tests of simulated underground lignite and hard coal gasification in the ex situ reactors were conducted to explore the environmental aspect of the UCG process in terms of amount and composition of wastewater produced in the process. After 2 h of lignite/hard coal ignition process, the burner was replaced with the gasification agents supply system and the gasification process was started. Pure oxygen was supplied to the reaction zone with a flow rate varying from 3 to 6 m^3^/h. The total gas and wastewater yield, calorific value of product gas as well as coal consumption rate and gross energy efficiency after the 12th, 24th, 36th and 48th hour of the ex situ UCG process duration are presented in Table 3.

**Table 3:** Total volume and selected balance parameters of the ex situ UCG wastewater

Parameter	Coal sample											
	BL				ZHC				BHC			
	UCG experiment duration [h]/Object no [−]											
	12/1	24/2	36/3	48/4	12/5	24/6	36/7	48/8	12/9	24/10	36/11	48/12
Wastewater production [kg]	65	62.5	52.5	47.5	31.25	45	36.25	17.5	11	15	13	7
Total gas production												
H_2_ [kg]	0.76	1.86	3.18	2.99	2.76	4.40	4.08	4.21	2.99	0.58	1.47	0.6
CO [kg]	5.73	11.06	18.95	13.17	45.22	62.16	71.21	79.66	55.36	33.34	92.22	43.15
CO_2_ [kg]	173.76	279.28	314.81	201.19	135.76	112.33	103.41	62.99	29.62	97.07	130.86	100.50
CH_4_ [kg]	0.64	1.58	2.66	2.36	3.11	3.69	3.00	3.34	1.53	0.05	0.14	0.05
C_2_H_6_ [kg]	0.03	0.06	0.08	0.12	0.24	0.28	0.25	0.28	0.01	0	0	0
H_2_S [kg]	0	0	0	0	0.32	0.94	1.00	0.87	0.02	0.28	0.47	0.29
O_2_ [kg]	13.91	5.97	5.28	3.17	16.43	2.78	2.99	2.38	10.20	0.37	0.68	0.39
N_2_ [kg]	8.38	13.37	14.64	9.66	7.88	7.78	7.56	5.84	29.47	2.37	3.72	2.45
Calorific value [MJ/m^3^]	1.54	2.22	3.10	3.87	6.07	8.06	8.25	9.75	7.94	8.36	6.99	5.43
Total coal consumption [kg]	102.5	167.3	195.59	89.6	86.4	88.37	89.69	79.25	48.5	59.7	96.3	58.7
Gross energy efficiency [%]	9.9	8.7	10.4	28.5	30.6	39.8	40.1	53.7	74.2	37.7	28.6	36.4

The total volume of wastewater produced after the 12th, 24th, 36th, and 48th hour of BL gasification and the total coal consumption were higher than those reported for ZHC and BHC. This may be attributed to the moisture content in the coal samples used, which is one of the crucial parameters determining gasification conditions, quality, and amounts of the products as well as suitability of coal for the UCG process. The tested ZHC and BHC were characterized by lower moisture content than BL (see Table 1). Higher moisture content in lignite block is also the main reason for lower calorific value of product gas and lower gross energy efficiency of the process in comparison with the respective values reported for hard coal ex situ UCG. In case of lignite gasification, the energy was partially consumed in water evaporation, which led to a decrease in the temperature in the reaction zone. This explains also the higher production of carbon dioxide in lignite gasification, when compared to hard coal gasification experiments. The total amount of wastewater generated decreased in time in all gasification tests. The lowest amount of wastewater was produced after the 48th hour of UCG process duration. Lignite was also characterized by lower carbon content and therefore the progress of the reaction zone was faster than in case of hard coal gasification experiments. The quality of lignite gasification product gas was relatively low in comparison with hard coal gasification tests. The average calorific value varied from 1.54 MJ/m^3^ in the first 12 h of process duration to 3.87 MJ/m^3^ between the 36th and 48th hour of the UCG experiment duration. The calorific value of product gas in ZHC and BHC gasification tests varied from 6.07 to 9.75 MJ/m^3^ after the 12th and 48th hour of the process duration and from 7.94 to 5.43 MJ/m^3^ after the 12th and 48th hour of the process duration, respectively.

**Fig. 2:**
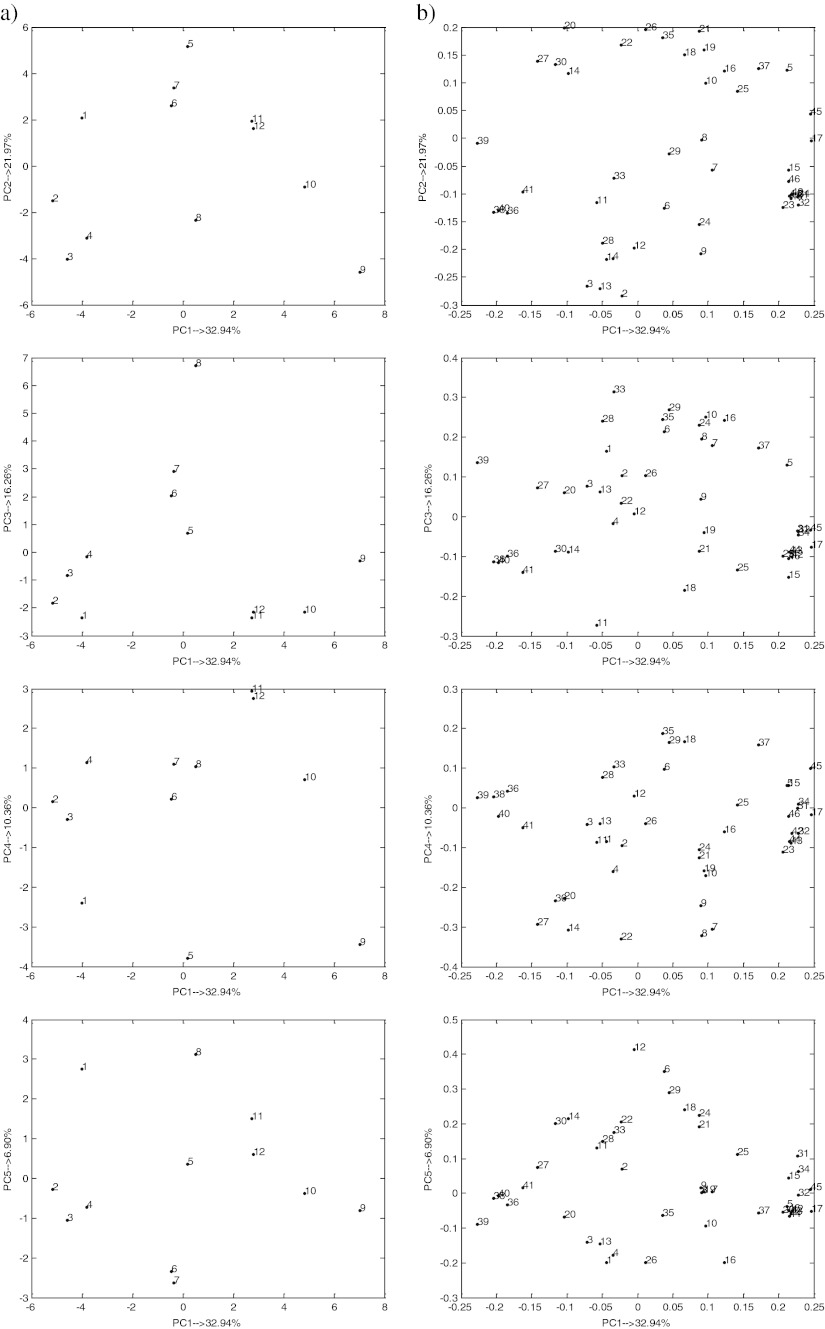
**a** Score plots and **b** loading plots as a result of PCA for centered and standardized data **X**(12 × 46)

The composition of wastewater varied in time, irrespective of coal type. The PCA method was applied to investigate the relationships between parameters of wastewater sampled after the 12th, 24th, 36th, and 48th hour of the experiments duration. The percent of modeled variance (Wold 1987) was applied in order to correctly determine the number of significant components (PCs). The reduction of data dimensionality was ineffective, since the PCA model with five PCs described 88.43 % of the total data variance. Score plots and loading plots obtained as the result of the PCA analysis are presented in Fig. 2.

The PC1 described 32.94 % of the total data variance. It showed the differences between the samples taken in the entire period of experiment duration. The most significant difference was observed between the wastewater sampled after the 24th hour of BL gasification experiment (object no. 2) and after the 12th hour of BHC gasification test (object no. 9). Along the PC1, three groups of wastewater sampled in BL (objects nos. 1–4), ZHC (objects nos. 5–8), and BHC (objects nos. 9–12) gasification experiments, respectively, may be distinguished. Based on the loading plots, a conclusion may be drawn that the differences between the samples collected in lignite and hard coal gasification experiments were caused mainly by the highest anthracene content (parameter no. 39) observed in lignite UCG experiment (objects nos. 1–4). The wastewater generated in hard coal gasification experiments was characterized by relatively higher chromium and benzo(*k*)fluoranthene contents (parameter nos. 17 and 45). The highest values of these parameters were observed for BHC gasification samples (objects nos. 9–12). The uniqueness of the sample collected after the 12th hour of BHC gasification test resulted from the highest chromium content (parameter no. 17) and the lowest anthracene content (parameter no. 39). The uniqueness of the sample collected after the 24th hour in BL gasification test stemmed from high anthracene content (parameter no. 39).

The PC2, which described 21.9 % of the data variance was constructed mainly due to the difference between the samples collected after the 12th hour of ZHC gasification (object no. 5) and after the 36th hour of BL and BHC gasification experiments (objects nos. 3 and 9), respectively. Based on the loading plot, a conclusion may be drawn that the sample collected after the 12th hour of ZHC gasification experiment was characterized by relatively high cadmium, lead, and naphthalene contents (parameter nos. 20, 26, and 35) and the highest cobalt content (parameter no. 21). Objects nos. 3 and 9 were characterized by relatively high pH, and high ammonia nitrogen and ammonium content (parameter nos. 2, 3, and 13) and low cadmium, cobalt, lead, and naphthalene contents (parameter nos. 20, 21, 26, and 35). The highest ammonia nitrogen and ammonium contents (parameter nos. 3 and 13) and the lowest manganese content (parameter no. 22) were observed for the sample collected after the 36th hour of BL gasification experiment (object no. 3).

Based on the PC3 (describing 16.26 % of the data variance) the uniqueness of wastewater sampled after the 48th hour of ZHC gasification test (object no. 8) was observed. The sample was characterized by the highest ethyl benzene content (parameter no. 33) and the lowest sulfates content (parameter no. 11). The PC4, which described 10.36 % of the total variance was constructed due to the difference between the samples produced after the 12th and 24th hour of BHC (objects nos. 11 and 12) and after the 12th and 36th hour of ZHC and BHC gasification experiments (objects nos. 5 and 9), respectively. The PC5 revealed the uniqueness of wastewater sampled after the 12th hour of BL gasification experiment and after the 24th, 36th, and 48th hour of ZHC gasification (objects nos. 1, 6, 7 and 8, respectively). Based on the loading plot, a conclusion may be drawn that the uniqueness of the samples produced after the 12th and 48th hour of BL and ZHC gasification experiments (objects nos. 1 and 8, respectively) was caused by the highest total amount of chloride and sulfate (parameter no. 12) when compared to all the remaining samples. The samples collected after the 24th and 36th hour of ZHC gasification (objects nos. 6 and 7) were characterized by the lowest total chlorides and sulfate amounts (parameter no. 12), the highest boron content (parameter no. 16) and relatively high conductivity and lead content (parameter nos. 1 and 26) when compared to the remaining samples. The uniqueness of the sample produced after the 36th hour of ZHC gasification (object no. 7) was caused by the highest lead content (parameter no. 26).

Since the compression of the data using PCA was ineffective, a conclusion may be drawn that only weak correlation between the variables could be observed in the presented data set. The loading plots revealed a positive correlation between pH, ammonia nitrogen, and ammonium (parameter nos. 2, 3, and 13); lead and naphthalene (parameter nos. 26 and 35); acenaphthene, phenanthrene, and fluoranthene (parameter nos. 36, 38, and 40); and copper, benzene, toluene, xylene, benzo(*a*)anthracene, chrysene, and benzo(*b*)fluoranthene contents (parameter nos. 23, 31, 32, 34, 42, 43, and 44). Furthermore, a negative correlation was observed between ammonia nitrogen (parameter no. 3) and cobalt and naphthalene contents (parameter nos. 21 and 35), between pH (parameter no. 2) and lead and naphthalene contents (parameter nos. 26 and 35), between ammonium (parameter no. 13) and cobalt and naphthalene contents (parameter nos. 21 and 35) and between anthracene (parameter no. 39) and arsenic and benzo(*a*)pyrene contents (parameter nos. 15 and 46).

**Fig. 3:**
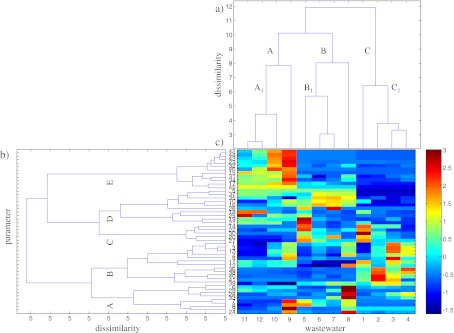
The dendrograms of **a** wastewater samples (*objects*) in the space of the 46 measured parameters (listed in Table 2) and **b** parameters in the space of 12 objects by the Ward linkage method using Euclidean distance as the similarity measure with **c** a color map of the studied data sorted according to the Ward linkage method

Efficient compression of the studied wastewater data was not possible with application of the PCA, as a standard method of data exploration, and obtained results required investigation of many two-dimensional plots. All the detailed conclusions presented above allowed extracting only general information on the analyzed wastewater sampled in lignite and hard coal ex situ UCG experiments. The hierarchical clustering method was applied to explore the studied data set and to examine the similarities between the samples. This method helped to reveal the internal data structure and, thereof, its clustering tendency. The results presented in Fig. 3 were based on the Euclidean distance and the Ward linkage algorithm.

The dendrogram presented in Fig. 3a allows revealing three clusters: the cluster A grouping samples obtained after the 12th, 24th, 36th, and 48th hour of BHC gasification experiment (objects nos. 9–12), the cluster B, composed of samples of ZHC gasification test (objects nos. 5–8) and the cluster C including samples of BL gasification (objects nos. 1–4). In the main clusters, additional sub-clusters can be distinguished. Sub-clusters A1 (objects nos. 10–12) and C1 (objects nos. 2–4) group samples collected after the 24th, 36th, and 48th hour of BHC and BL gasification experiments, respectively. Sub-cluster B1 (objects nos. 5–7) is composed of wastewater sampled after the 12th, 24th and 36th hour of ZHC gasification test. The dendrogram constructed for the measured parameters (see Fig. 3b) revealed five main groups:

- Group A, composed of parameter nos. 6–9, 24, 28, 29, and 33, which represent contents of chlorides, total cyanides, free cyanides, bound cyanides, molybdenum, selenium, titanium, and ethyl benzene, respectively,
- Group B, including parameter nos. 1–4, 11–13, 36, and 38–41, which represent conductivity, pH, ammonia nitrogen, suspended matter, sulfates, total amount of chloride and sulfate, ammonium, acenaphthene, phenanthrene, anthracene, fluoranthene, and pyrene contents, respectively,
- Group C, collecting parameter nos. 14, 20, 22, 27, and 30, which represent antimony, cadmium, manganese, mercury and iron contents, respectively,
- Group D, composed of parameter nos. 5, 10, 16, 18, 19, 21, 25, 26, 35, and 37, which represent contents of nitrites, phenols, boron, zinc, aluminum, cobalt, nickel, lead, naphthalene, and fluorene, respectively, and
- Group E, including parameter nos. 15, 17, 23, 31, 32, 34, and 42–46, which represent contents of arsenic, chromium, copper, benzene, toluene, xylene, benzo(*a*) anthracene, chrysene, benzo(*b*)fluoranthene, benzo(*k*)fluoranthene, and benzo(*a*)pyrene contents, respectively.

The dendrogram showed in Fig. 3a presents the data structure, but does not enable investigation of the observed patterns in terms of the original parameters. To solve this problem, HCA could be complemented with a color map of experimental data sorted according to the specific order of objects and parameters (Smoliński et al. 2002).

The HCA completed with a color map of the standardized experimental data is a simple visualization method. Namely, the color map is constructed for standardized transposed data matrix **X**_c_. The objects (wastewater samples) and parameters are sorted according to the order of objects and parameters at dendrograms in Fig. 3a and b. The image with pixels represents the matrix elements. The neighboring objects (wastewater samples) and parameters are ordered according to their similarity.

The analysis of the dendrogram of 12 objects in the space of the 46 measured parameters sorted according to the Ward linkage method with the color data map (see Fig. 3c) enables more in-depth investigation of the resulting clustering tree. The wastewater samples of the cluster A (objects nos. 9–12) were characterized by higher contents of arsenic, chromium, benzene, xylene, and benzo(*k*)fluoranthene (parameter nos. 15, 17, 31, 34, and 45) and lower contents of cadmium, mercury, and anthracene (parameter nos. 20, 27, and 39) than observed for the remaining samples.

Furthermore, the sample collected after the 12th hour of BHC gasification (object no. 9) was characterized by the highest contents of total and bound cyanides, arsenic, chromium, copper, benzene, toluene, xylene, benzo(*a*) anthracene, chrysene, and benzo(*b*)fluoranthene (parameter nos. 7, 9, 15, 17, 23, 31, 32, 34, and 42–44), and the lowest content of titanium (parameter no. 29).

All samples of ZHC gasification process grouped in the cluster B were characterized by relatively lower content of sulfates (parameter no. 11), and higher contents of phenols and boron (parameter nos. 10 and 16), than observed for the remaining samples of BHC and BL gasification experiments.

The sample collected after the 48th hour of ZHC gasification test (object no. 8) differed from the remaining samples collected after the 12th, 24th, and 36th hour of process duration (objects nos. 5–7) due to the highest content of chlorides, total amount of chloride and sulfate, copper, molybdenum, selenium, and titanium (parameter nos. 6, 12, 23, 24, 28, and 29). The uniqueness of the sample collected after 12th hour of ZHC gasification (object no. 5) was also observed resulting from high contents of free and bound cyanides, antimony, cadmium, and mercury (parameter nos. 8, 9, 14, 20, and 27), the highest contents of aluminum, cobalt and manganese (parameter nos. 19, 21, and 22) as well as the lowest contents of acenaphthene, phenanthrene, and fluoranthene (parameter nos. 36, 38, and 40).

All samples of BL experiments grouped in the cluster C differ from the samples of hard coal gasification experiments mainly due to relatively lower contents of nitrites, boron, chromium, aluminum, lead, benzene, toluene, xylene, naphthalene, fluorene, benzo(*a*) anthracene, chrysene, benzo(*b*)fluoranthene, benzo(*k*)fluoranthene, and benzo(*a*)pyrene (parameter nos. 5, 16, 17, 19, 26, 31, 32, 34, 35, 37, and 42–46) and relatively higher contents of acenaphthene, phenanthrene, fluoranthene, and pyrene (parameter nos. 36, 38, 40, and 41).

The sub-cluster C1 (objects nos. 2–4) was characterized by the highest contents of acenaphthene, phenanthrene, fluoranthene, and pyrene (parameter nos. 36, 38, 40, and 41). The sample collected after the 36th hour of lignite gasification (object no.3) was characterized by the highest conductivity (parameter no.1) and the highest contents of ammonia nitrogen, suspended matter, ammonium, and anthracene (parameter nos. 3, 4, 13, and 39), whereas the wastewater sampled after 48th hour of lignite gasification (object no. 4) was characterized by the highest content of acenaphthene (parameter no. 36) and the lowest contents of antimony, boron, zinc, and pyrene (parameter nos. 14, 16, 18, and 41). The uniqueness of the sample collected after the 12th hour of lignite gasification (object no. 1) resulted from the highest contents of sulfates and iron (parameter nos. 11 and 30), relatively high contents of total amount of chloride and sulfate, antimony, cadmium, manganese and mercury (parameter nos. 12, 14, 20, 22, and 27) and the lowest contents of ammonia nitrogen, ammonium and chromium (parameter nos. 3, 13, and 17).

**Fig. 4:**
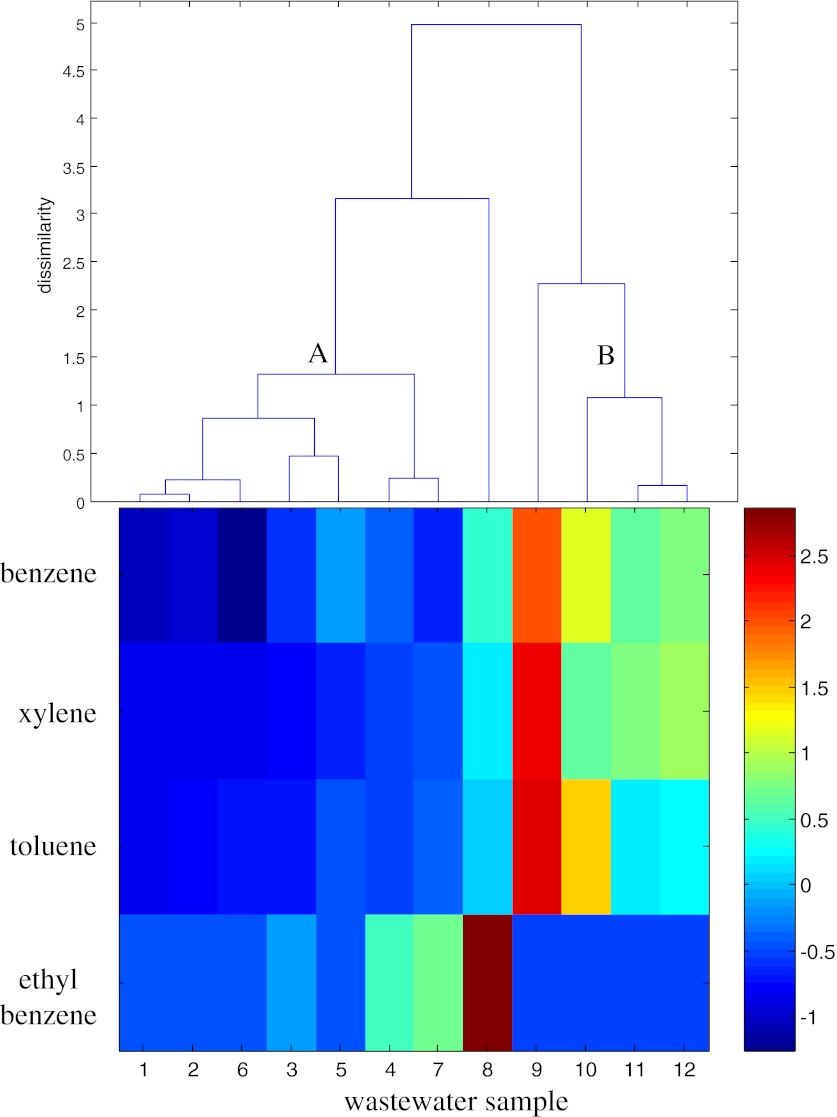
Dendrogram of wastewater samples (*objects*) with visual complement in the space of the BTEX parameters (benzene, toluene, ethyl benzene, and xylene)

**Fig. 5:**
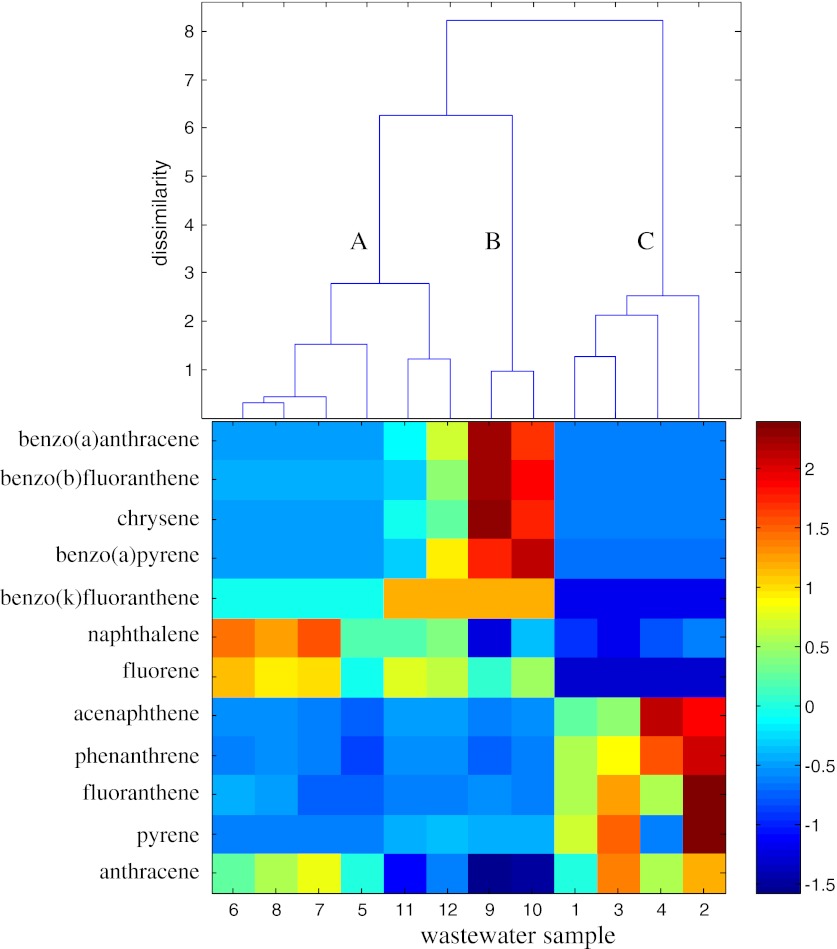
Dendrogram of wastewater samples (*objects*) with visual complement in the space of the PAHs parameters

In addition to water-soluble, polar phenolics, low soluble organic compounds such as BTEX (parameter nos. 31–34) as well as the PAHs (parameter nos. 35–46) constitute the major threat to the groundwater in terms of UCG induced contamination. PAHs are one of the most widespread organic pollutants which could be formed in underground coal gasification. The amount and type of PAHs as well as their homologs depend on the operating conditions of combustion/gasification process. The PAHs toxicity is structurally dependent (Collins et al. 1998). The BTEX constitute a serious threat to the underground water system for their strong and long term toxic effects. Benzene is also known as a severe carcinogen. Clustering of the wastewater samples in the BTEX and in the PAHs parameter space is presented in Figs. 4 and 5, respectively.

Two clusters of samples in the BTEX space differed from the clusters in the entire space of 46 measured parameters. The cluster A is composed of the BL gasification samples (objects nos. 1–4) and the samples obtained after the 12th, 24th, and 36th hour of ZHC gasification experiments (objects nos. 5–7). The cluster B is composed of the samples collected after the 24th, 36th, and 48th hour of BHC gasification (objects nos. 10–12) (see Fig. 4). The uniqueness of the samples collected after the 48th and 12th hour of ZHC and BHC gasification experiments (objects nos. 8 and 9), respectively, could be also be observed. The samples of the cluster A were characterized by relatively lower contents of benzene, toluene and xylene when compared to the samples of the cluster B. The uniqueness of the sample collected after the 48th hour of ZHC gasification (object no. 8) resulted from the highest content of ethyl benzene, whereas the uniqueness of the wastewater sampled after the 12th hour of BHC gasification (object no. 9) was caused by the highest contents of benzene, toluene, and xylene.

The samples of ZHC gasification experiment (objects nos. 5–8) and the samples collected after the 36th and 48th hour of BHC gasification (objects nos. 11 and 12) included in the cluster A were characterized by relatively higher naphthalene and fluorene contents. The uniqueness of the objects nos. 6–7 of the cluster A could be also observed. These objects were characterized by high anthracene content, the highest naphthalene and fluorene contents and the lowest pyrene content.

The samples collected after the 12th and 24th hour of BHC gasification (objects nos. 9 and 10) grouped in the cluster B were unique due to high benzo(*k*)fluoranthene, the highest benzo(*a*)anthracene, chrysene, benzo(*b*)fluoranthene, and benzo(*a*)pyrene and the lowest anthracene contents when compared to the remaining wastewater samples.

The cluster C, composed of samples collected during the entire BL gasification experiment (objects nos. 1–4) was characterized by the lowest naphthalene, fluorene, and benzo(*k*)fluoranthene contents and relatively higher acenaphthene, phenanthrene, anthracene, fluoranthene, and pyrene contents. The uniqueness of the wastewater sampled after the 24th and 48th hour of lignite gasification (object no. 2) was caused by the highest phenanthrene, fluoranthene, and pyrene contents and the highest acenaphthene content, respectively.

## Conclusions

1. The wastewater generated in the UCG poses the potential threat to the environment if treated inappropriately, since it contains high concentrations of inorganic and organic (phenolics, PAHs, BTEX) components.
2. Lignite ex situ UCG process was characterized by higher total amount of wastewater generated and higher coal consumption rate, when compared to the respective values reported for the ex situ UCG process on hard coal.
3. Higher amount of wastewater generated in lignite gasification process resulted from significantly higher moisture content in lignite when compared to hard coal samples.
4. The chemometric methods, such as the PCA and the HCA enabled to effectively explore environmental data concerning the amounts and composition of wastewater generated in lignite and hard coal ex situ UCG experiments.
5. The samples collected in lignite gasification experiment were characterized by the highest acenaphthene, phenanthrene, anthracene, fluoranthene, and pyrene contents, whereas the samples of hard coal gasification experiments were characterized by relatively higher nitrites, arsenic, chromium, copper, benzene, toluene, xylene, benzo(*a*)anthracene, chrysene, benzo(*b*)fluoranthene, benzo(*k*)fluoranthene, and benzo(*a*)pyrene contents.
6. Apart from phenolics, PAHs were identified as the most widespread organic pollutants which could be formed in the process of the underground coal gasification. The amount and types of PAHs as well as their homologs depend on the type of the gasification process. The BTEX group creates a serious threat to the underground water system for their strong and long term toxic effects.
7. Clustering of the wastewater samples in the BTEX and in the PAHs parameters space revealed the differences between the tested wastewater samples.
8. The uniqueness of the samples collected after the 24th, 36th and 48th hour of BHC gasification resulted from relatively higher benzene, toluene and xylene contents, when compared to the samples of BL and ZHC gasification.
9. The samples collected after the 12th and 24th hour of BHC gasification were also characterized by high benzo(*k*)fluoranthene content, the highest benzo(*a*)anthracene, chrysene, benzo(*b*)fluoranthene, and benzo(*a*)pyrene contents and the lowest anthracene content.
10. The uniqueness of the sample collected after the 48th hour of ZHC gasification resulted from the highest content of ethyl benzene and the uniqueness of the wastewater sampled after the 12th hour of BHC gasification could be attributed to the highest contents of benzene, toluene, and xylene.
